# Vital Statistics

**Published:** 1887-03

**Authors:** 


### VITAL STATISTICS.

Our legislators evidently have no conception of the value or importance of vital statistics;—inasmuch as every bill looking to the creation of a State Board of Health which has been presented, has been defeated. We do not propose here, and now, to enumerate the advantages to the public—in every way—to be derived from carefully preserved vital statistics; it is to be presumed that our readers understand and appreciate them, and know that it is only possible to see the necessity for reforms in habit, modes of life, occupation, etc.,—and to bring them about, by keeping a careful record of deaths, and the causes. We presume, also, that our readers are familiar with the circumstances which finally led to the passage of the first, now famous, Public Health Bill, in Great Britain. Dr. Farr spent his valuable life in the laborious compilation of statistics of deaths; and ascertaining the causes of the larger number to be unsanitary dwellings and occupations,—procured the passage of this bill. Under its provisions such reforms were instituted as lead, in seven years, to actually adding some two and a half, or three years to the life of every man, woman and child in the United Kingdom.

Many of the diseases of which people die in Texas are preventable. It only remains to tabulate the deaths, and in time, as certain these causes—when, the causes removed, the number of deaths will be diminished.

The above is suggested on reading the action of the Johnson County Board of Censors. Dr. Osborn is entitled to be called the Farr of this State; he is the pioneer. He sees, and realizes the fact, that the hope of assistance in this matter by the State, is Utopian, and knowing, also, the necessity of registration, he proposes a way in which it can be done independently of the State, i. e., by each county, through the local medical society. We commend the example to our county societies, and suggest that they institute similar proceedings. The JOURNAL cheerfully places its columns at their disposal, for the publication of their work.

Meantime we would suggest that it is possible, through the efforts of individual members of these local societies, to so impress Representatives and Senators with the value, and necessity, of registration;—and by showing them the efforts being made by those societies—that in time the whole Legislature may be brought to comprehend the situation; then State provision will be made. It is almost *sinful* that such valuable statistics are not now being preserved. Johnson county is certainly the pioneer in these good works; from that county emanated the only meteorological reports yet published by the JOURNAL, with an article on "The Relation of Areas of Low Barometer to Epidemics," and we are rather surprised it did not attract more attention in the State; it was well received in other States. We bid our Johnson county friends persevere, and hope they will not be disappointed by the cold water of seeming indifference to their labors; the fruits will come by and bye. To our mind, the duty on the part of the State to protect the people from sickness, and from ignorant practitioners, is as imperative as it is to protect them from any other source, or kind of danger; yet the Legislature cannot be made to see it in that light, until the profession, united into one harmonious body, speak the sentiment in no uncertain tones. Property in Texas is hedged round about with every kind of safeguard that ingenuity and selfish interest can suggest; but life and health are left to take care of themselves.

